# Cytokines Orchestrating the Natural Killer-Myeloid Cell Crosstalk in the Tumor Microenvironment: Implications for Natural Killer Cell-Based Cancer Immunotherapy

**DOI:** 10.3389/fimmu.2020.621225

**Published:** 2021-01-29

**Authors:** Silvia Gaggero, Kristina Witt, Mattias Carlsten, Suman Mitra

**Affiliations:** ^1^Univ. Lille, CNRS, INSERM, CHU Lille, Institut pour la Recherche contre le Cancer de Lille, UMR9020 – UMR-S 1277 – CANTHER - Cancer Heterogeneity, Plasticity and Resistance to Therapies, Lille, France; ^2^Center for Hematology and Regenerative Medicine, Department of Medicine, Huddinge, Karolinska Institutet, Stockholm, Sweden; ^3^Center for Cell Therapy and Allogeneic Stem Cell Transplantation, Karolinska University Hospital, Stockholm, Sweden

**Keywords:** natural killer (NK) cells, myeloid cells, cytokines, tumor microenvironment, cancer immunotherapy, tumor immunity

## Abstract

Natural killer (NK) cells are endowed with germline-encoded receptors that enable them to detect and kill malignant cells without prior priming. Over the years, overwhelming evidence has identified an essential role for NK cells in tumor immune surveillance. More recently, clinical trials have also highlighted their potential in therapeutic settings. Yet, data show that NK cells can be dysregulated within the tumor microenvironment (TME), rendering them ineffective in eradicating the cancer cells. This has been attributed to immune suppressive factors, including the tumor cells *per se*, stromal cells, regulatory T cells, and soluble factors such as reactive oxygen species and cytokines. However, the TME also hosts myeloid cells such as dendritic cells, macrophages, neutrophils, and myeloid-derived suppressor cells that influence NK cell function. Although the NK-myeloid cell crosstalk can promote anti-tumor responses, myeloid cells in the TME often dysregulate NK cells *via* direct cell-to-cell interactions down-regulating key NK cell receptors, depletion of nutrients and growth factors required for NK cell growth, and secretion of metabolites, chemokines and cytokines that ultimately alter NK cell trafficking, survival, and cytotoxicity. Here, we review the complex functions of myeloid-derived cytokines in both supporting and suppressing NK cells in the TME and how NK cell-derived cytokines can influence myeloid subsets. We discuss challenges related to these interactions in unleashing the full potential of endogenous and adoptively infused NK cells. Finally, we present strategies aiming at improving NK cell-based cancer immunotherapies *via* pathways that are involved in the NK-myeloid cell crosstalk in the TME.

## Introduction

Natural Killer (NK) cells are cytotoxic lymphocytes that innately recognize their target cells based on signals from an array of germline-encoded inhibitory and activating cell surface receptors (1). While inhibition is mainly mediated by HLA class I-binding receptors such as KIR, LIR-1, and NKG2A, activation is triggered by the NKG2D, DNAM-1, NKp30, NKp46, and 2B4 receptors, among others (2). Experimental approaches delineating how NK cells target tumor cells have in more recent years been harmonized with studies evidencing their role in tumor immune surveillance (3) and clinical therapy to treat patients with cancer (4, 5). However, it has also become increasingly clear that NK cells are often dysfunctional in cancer patients (6, 7). This is most prominent in the tumor microenvironment (TME), although also observed in blood and other tissues in patients with advanced cancer (6–8). Factors suppressing endogenous or adoptively infused NK cells in the TME are likely limiting the full potential of NK cell-based cancer immunotherapies.

NK cells can be disarmed in the TME by both direct and indirect interactions with the tumor cells (6, 7, 9). However, several other cell types in the TME, such as stroma cells and immune cells, acting by direct interactions and *via* release of reactive oxygen species, growth factors, and cytokines, can also induce NK cell dysfunction (9–11). Both the degree and mode of NK cell suppression in the TME may dynamically vary from early to later stages of cancer development as well as between different tumor histotypes. Beyond reducing the anti-tumor cytotoxicity of NK cells *per se*, suppression of NK cells in the TME can also negatively impact their ability to recruit other immune cells (12–15), which is crucial for initiating and maintaining proper anti-tumor responses. In this regard, a pivotal interaction in the TME is the one between NK cells and myeloid cells, such as dendritic cells (DCs), macrophages, neutrophils, and myeloid-derived suppressor cells (MDSCs) (16–19). While NK cells positively promote DC infiltration and maturation *via* release of pro-inflammatory cytokines such as interferon (IFN)-*γ* (20), myeloid-derived cytokines, including interleukin (IL)-12, IL-15, and IL-18, critically promote NK cell maturation, proliferation, and anti-tumor functions (21). However, aggravation of the TME often observed in more advanced stages of cancer direct myeloid cells towards a suppressive phenotype that instead can impede NK cell functions *via* secretion of cytokines, such as transforming growth factor (TGF)-β, IL-1β, and IL-10 (22–24). Thus, the NK-myeloid crosstalk is intricate but critical for proper anti-tumor properties of NK cells in the TME.

Here we give an overview of the cytokines involved in the interplay between NK cells and myeloid cells in the general TME. We discuss how myeloid cells promote NK cell functions and vice versa, but foremost, how this interaction can hinder NK cell-mediated tumor rejection. We outline current methods and possible future approaches to enhance anti-tumor responses by NK cells *via* administration or manipulation of cytokines and cytokine signaling, as well as preventing myeloid cell infiltration into the TME. This review highlights that a better understanding of the crosstalk between myeloid cells and NK cells is likely critical to improve the efficacy of NK cell-based cancer immunotherapy.

## Myeloid-Derived Cytokines Promoting Natural Killer Cell Responses to Cancer

**Figure 1:**
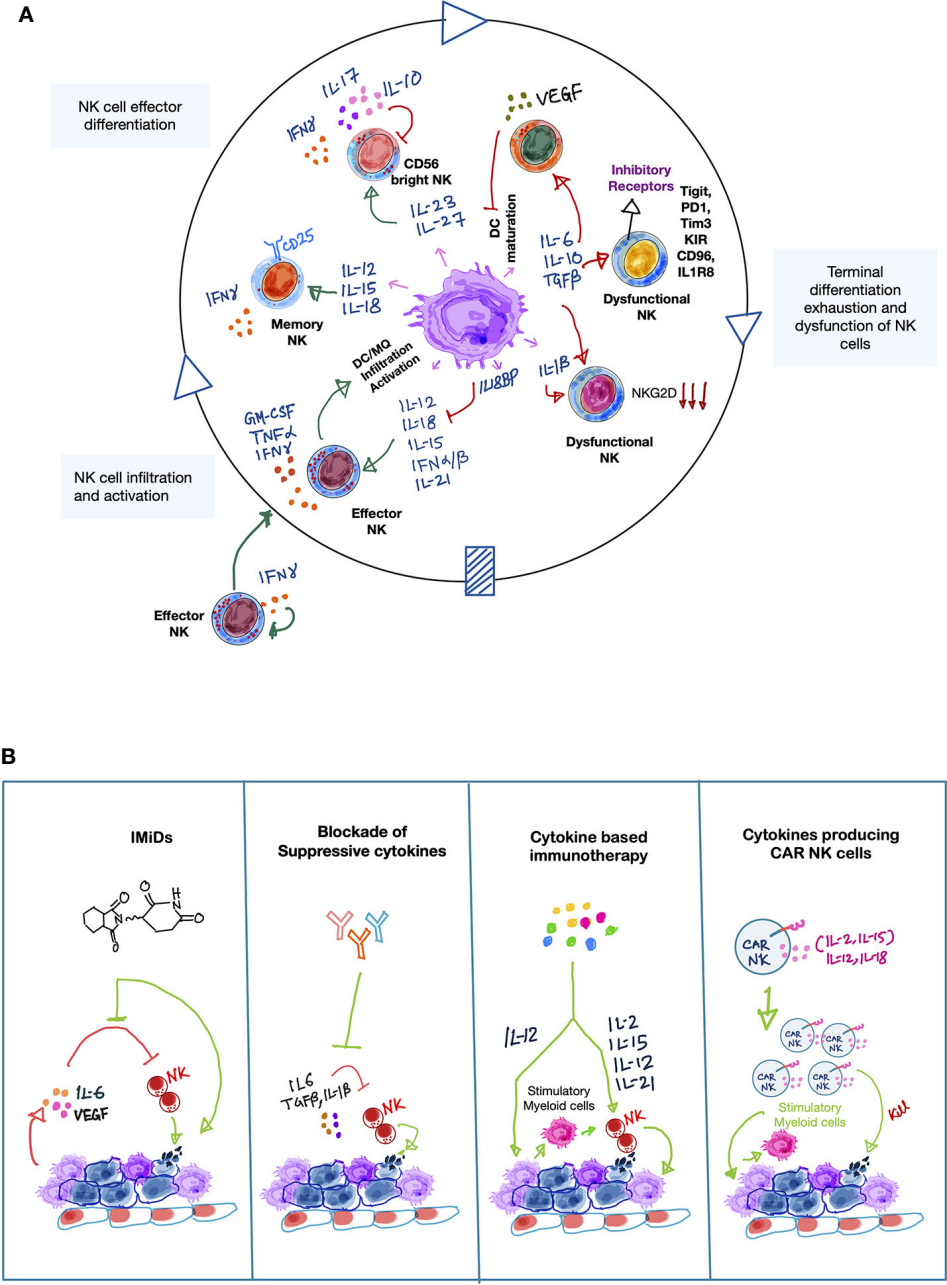
Cytokines involved in the NK-myeloid cell cross talk in the tumor microenvironment. **(A)** A simplified schematic illustration showing the orchestra of myeloid- and NK cell-derived cytokines involved in forming anti-tumor immune responses by NK cells in the tumor microenvironment (TME). Activated NK cells produce IFN-γ which indirectly promotes recruitment of other NK cells from peripheral blood to the tumor sites. Upon recognition of tumor antigens, myeloid cells, especially DCs and macrophages produce inflammatory cytokines such as type-1 IFNs, IL-12, IL-15, IL-18, IL-21. These cytokines either alone or cooperatively promote NK cell survival, proliferation, maturation and production of spectrum of pro-inflammatory cytokines, including IFN-γ, TNF-α, GM-CSF, which further boost anti-tumor immune-activating potential of myeloid cells while recruiting additional inflammatory myeloid cells [including M1 macrophages (MQ), mature dendritic cells (DCs)] to sustain the anti-tumor immune response. Furthermore, myeloid-derived IL-23 and IL-27 cytokines can also promote NK cell activity by inducing IFN-γ production, but they can also negatively influence the NK-myeloid cell anti-tumor crosstalk by secretion of tumor-promoting cytokines such as IL-17 and IL-10, respectively. This suggests a dual role of IL-23 and IL-27 in NK cell-mediated tumor immunity. In line with the dual roles of certain cytokines, myeloid cells can become immune suppressive (myeloid-derived suppressor cells; MDSCs). This is frequently occurring during cancer progression. MDSCs secrete a plethora of immune suppressive cytokines that negatively influence the anti-tumor potential of NK cells *per se* but also impair anti-tumor responses normally resulting from the NK-myeloid cell crosstalk. For example, suppressive cytokines promote NK cell exhaustion and directly impair NK cell-mediated cytolytic activity, while limiting the ability of myeloid cells to produce NK cell stimulatory cytokines such as IL-12, IL-15, and IL-18. Green arrows indicate positive interactions and red arrows indicate negative interactions. **(B)** Therapeutic approaches that directly or indirectly modulate cytokine mediators that enhance NK cell-mediated anti-cancer responses in the TME.** **Simplified illustrations showing validated therapeutic approaches that can either restore or reinforce a stimulatory cytokine environment to augment NK cell-mediated tumor killing activity. On one hand, immunomodulatory drugs (IMiDs) such as lenalidomide can indirectly augment NK cell anti-tumor activity by reducing the levels of pro-tumorigenic factors, such as IL-6 and VEGF, while stimulating other immune cells to secrete IL-2. Accordingly, targeted blockade of immune-suppressive cytokines, such as IL-6, TGF-β can also positively impact NK-myeloid anti-tumor cross-talk in a similar manner. On the other hand, recombinant or synthetic cytokines as well as cell-based therapies such as cytokine-secreting CAR-NK cells can directly influence NK cell-mediated cancer cell killing. Importantly, cytokine-activated NK cells can further edit myeloid cells to enhance anti-tumor response *via* the production of inflammatory cytokines, such as IFN-γ. Light green arrows show the mode of therapeutic action.

Several myeloid cells, exemplified by macrophages and DCs, are characterized by a pro-inflammatory phenotype and release cytokines such as type-1 IFNs, IL-12, IL-15, IL-18, IL-21 (**Table 1A**) upon recognition of damage-associated molecular patterns (DAMPs) on the transformed cells (70, 71). This pro-inflammatory cytokine milieu, together with key chemokines, aid in the recruitment of NK cells to the tumor site while promoting their persistence and anti-tumor effector functions (70, 72). Several of these cytokines have overlapping functions but also possess specific functions in the regulation of NK cell responses in cancer (35, 73–79) (**Figure 1** and **Table 1A**). In this section, we will present the key cytokines released by myeloid cells that promote anti-tumor cytotoxicity by NK cells but also give examples of how cytokines can have dual functions.

**Table 1:** Cytokines and chemokines involved in the NK-myeloid cell crosstalk and drugs directed to modulate these interactions.

A. Myeloid cell-derived cytokines and their effects on NK cells.					
Cytokine	Produced by	Effects	Therapy		Ref.
TGF-β	MDSCs, TAMs, tumor cells, mast cells	↓activating receptor, cytokine production, cytotoxicity, proliferation	Fresolimumab, galunisertib, M7824 (clinical trial)		(25–30)
IL-10	MDSCs, TAMs, NK cells, DCs, macrophages	↓/↑ cytotoxicity, cytokine production	–		(24, 31–33)
IL-32α	DCs	↓perforin, granzyme B	–		(34)
TNF	Macrophages	↑ cytokine production	–		(35)
IL-12	DCs, macrophages, monocytes, neutrophils	↑ cytotoxicity, cytokine production, proliferation, survival	IL-12, IL-12 + pembrolizumab (clinical trial)		(21, 36)
IL-15	DCs, macrophages, monocytes	↑ cytotoxicity, cytokine production, proliferation, survival, activating receptors, KIR	ALT-803 (phase 1 and 2 clinical trial), IL-15 + Ipilimumab and Nivolumab (phase 1 clinical trial)		(37–39)
IL-18	M0 macrophages, TAMs, DCs,	↑ cytotoxicity, cytokine production, proliferation, survival	IL-18 (phase 1 and 2 clinical trial)		(40–44)
IL-21	DCs	↑ cytotoxicity, proliferation, activating receptors	IL-21, IL-21 + Ipilimumab and nivolumab (phase-I and –II clinical trial)		(45)
IL-6	MDSC, TAM, tumor cell, macrophages, monocytes, mast cells	↓/↑ cytotoxicity, ↓ cytokine production	Tocilizumab (clinical trial)		(46, 47)
IL-1α	Monocytes, DCs, macrophages	↓maturation	Anakinra, Canakinumab, Isunakinra (phase 1 and 2 clinical trial)		(23)
IL-27	DCs, macrophages, MDSCs	↓/↑ cytotoxicity, cytokine production	p28 peptide		(48–53)
IL-23	MDSC and TAM, DC and macrophage	↓/↑ cytotoxicity, cytokine production	–		(54, 55)
IL-17	Neutrophils	↑cytotoxicity, ↓maturation	–		(56, 57)
IFN-α/β	DC	↑ cytotoxicity, cytokine production, proliferation, survival, NKG2D	IFN-α/β approved		(58)
B. NK cell-derived cytokines and their effects on myeloid cells.					
Cytokine	Target population	Effects		Therapy	Ref.
IFN-γ	DCs	maturation, activation		–	(59–61)
	TAMs	polarization towards pro-inflammatory Mφ		–	(62, 63)
	TANs	inhibition of pro-tumorigenic TANs		–	(64)
TNF-α	DCs	maturation, activation		–	(59–61)
	TAMs	polarization towards pro-inflammatory Mφ		–	(62)
HMGB1	DCs	activation		–	(43, 65)
GM-CSF	DCs	activation		–	(62)
	TAMs	polarization towards pro-inflammatory Mφ		–	(62)
	TANs	activation, promotes NETs		–	(66–68)
VEGF-A	Endothelial cells, tumor cells	proliferation, migration		–	(69)

A) Effects of myeloid cell-derived cytokines on NK cells and therapeutic targeting thereof. B) Effects of NK cell-derived cytokines on the maturation and differentiation of different myeloid cell subsets and therapeutic targeting thereof.

### Direct and Indirect Contribution of Interferon-α and Interferon-β in Natural Killer Cell Activation

The type-1 IFNs, IFN-α, and IFN-β are secreted by activated myeloid cells and stimulate NK cell expansion while enhancing the effector functions upon stimulation of the IFN-α receptor (IFNAR) (80, 81). Inversely, as highlighted in experimental models using IFNAR-deficient NK cells and NK cells with defective downstream signaling molecule transducer and activator-1 (STAT1), impaired type-1 IFN signaling results in defective functional NK cell maturation and hampered anti-tumoricidal potential in sarcoma and lymphoma mouse models (82, 83). Importantly, while transient or intermittent type-1 IFN stimulation results in preferential phosphorylation of STAT4 than STAT1 and thereby increased IFN-γ production by NK cells promoting pro-inflammation, chronic stimulation coupled with increased levels of IFN-γ also bolster NK cell cytotoxicity (81, 84). This is due to increases basal levels of STAT1 protein expression following chronic IFN-γ stimulation that triggers preferential activation of STAT1 over STAT4 (81, 84). Additionally, autocrine type-1 IFN signaling in activated myeloid cells induces interleukin (IL)-15 production, which is a critical cytokine for NK cell development, proliferation and cytotoxic function (37, 85).

### Regulation of Natural Killer Cell Activation and Effector Function by Interleukin-12 Family of Cytokines

The IL-12 family of heterodimeric cytokines, including IL-12, IL-23, and IL-27, critically regulate NK cell activation and effector functions (**Figure 1A** and **Table 1A**). Phagocytic macrophages and DCs produce these cytokines following the recognition of DAMPs on dying tumor cells. Despite sharing sequence similarly, these cytokines uniquely modulate NK cell function.

#### Interleukin-12

IL-12 (p35 and p40 complex) signals by engaging the heterodimeric receptor complex of IL-12Rβ1 and IL-12Rβ2 subunits that are readily expressed by mature activated but not immature NK cells. In NK cells, IL-12 signaling principally mediates STAT4 phosphorylation that is essential for both IFN-γ and perforin expression, as observed both in human NK cells *in vitro* and in murine NK cells *in vivo* (21, 86–89). Accordingly, blockade of IL-12 diminishes DC-induced IFN-γ production by NK cells, suggesting IL-12 is critical for optimal IFN-γ release by activated human NK cells (90). Additionally, IL-12 can work in concert with IL-15 and IL-18 to generate ‘memory-like’ NK cells, partly facilitated by epigenetic reprogramming of the CNS1 enhancer region of the *Ifng* locus in NK cells, which enables them to produce elevated levels of IFN-γ compared to conventional NK cells upon activation as shown by transferring IL-12/IL-15/IL-18-pretreated human NK cells in NSG mice (91).

#### Interleukin-23

IL-12p40 also interacts with p19 subunits forming the heterodimeric cytokine IL-23. Upon stimulation with IL-23, CD56^bright^ NK cells release higher levels of IFN-γ as compared to CD56^dim^ NK cells due to their higher expression levels of IL-23R. IL-23 also increases IL-18Rα expression, thus priming NK cells for IL-18-induced IFN-γ production. IL-23 stimulates human NK cell activation *in vitro* while inhibiting IL-15- and IL-18-induced NK cell proliferation (54). However, IL-23 reduces both cytotoxicity and IFN-*γ* production *in vivo*, indeed anti-IL-23 therapy synergizes with IL-2 or anti-erbB2 treatment in mammary and melanoma tumor models in an NK cell-dependent manner (55, 92).

#### Interleukin-27

The IL-27 heterodimer is composed of p28 and EBI3 and promotes pro- and anti-inflammatory functions mainly through STAT1 and STAT3 (93). Like IL-23, IL-27 shows differential activity on human CD56^bright^ and CD56^dim^ subsets, maybe related to the higher expression of IL-27Rα in the CD56^bright^ subset (48). While CD56^dim^ NK cells are not affected, CD56^bright^ NK cells acquire a regulatory phenotype. Yet, *in vivo* studies on a murine squamous cell carcinoma model and *in vitro* analysis on human NK cells also demonstrated that IL-27 primes NK cells to respond to IL-18, while inducing perforin, granzyme B, NKp46, and NKG2D expression, as well as promoting antibody-dependent cellular cytotoxicity (ADCC) (49–52, 94, 95).

### Critical Role of Gamma-Chain Cytokines Released by Myeloid Cells in Promoting Natural Killer Cell Survival, Proliferation, and Functions

The gamma-chain family consists of cytokines such as IL-2, IL-4, IL-7, IL-9, IL-15, and IL-21 that all bind the common gamma-chain receptor. Below we will focus the discussion on IL-15 and IL-21 that are commonly derived from myeloid cells.

#### Interleukin-15

IL-15/IL-5Rα induces NK cell survival and proliferation by acting as a soluble form or presented at the DC membrane. As mentioned above, it is also an essential driver of NK cell development and activation (37–39, 96). IL-15 expression correlates with NK cell infiltration in human tumor samples (97), and data from a murine melanoma model indicate CD11b^+^Ly6C^hi^Ly6G^-^ monocytic cells are the major source of this cytokine (78). IL-15, together with IL-12, also indirectly regulates NK cell functions by controlling metabolism *via* mTORC1 activation, which stimulates nutrient uptake, glycolysis, and OXPHOS, thereby providing the energy for NK cell proliferation, proper functions and enhanced persistance (36, 98). Interestingly, chronic IL-15 stimulation of NK cells results in exhaustion by reducing the mitochondrial respiratory capacity (99).

#### Interleukin-21

In addition to IL-15, DCs also release IL-21, which following STAT3 and STAT1 signaling in NK cells, promotes NK cell cytotoxicity *via* increased granzyme B and perforin expression. Moreover, STAT1 and PI3K pathways are essential for IL-21-mediated reversal of NK cell exhaustion in mice and for intratumoral human NK cells cultured *in vitro* (45). Interestingly, IL-21 differentially regulates the expression of activating receptors by inducing NKp30 levels while reducing NKG2D/DAP10 expression in human NK cell (100). However, IL-21 contributes to tumor rejection in an NKG2D-dependent manner in multiple mouse tumor models (101).

### Interleukin-17 Can Promote Natural Killer Cell Cytotoxicity While Limiting Terminal Differentiation of Natural Killer Cells

Neutrophils are the major producers of IL-17, which binds to a dimeric receptor and mainly signals through the NF-kB and ERK pathways (102). IL-17 has been shown to enhance NK cell recruitment in human esophageal cancer through tumor-derived chemokines and NK cell cytotoxicity through the increased expression of activating receptors, perforin, granzyme B, TNF-α, and IFN-*γ* (103). However, IL-17 has also been reported to limit IL-15-mediated terminal murine NK cell differentiation *via* upregulation of suppressor of cytokine signaling (SOCS), which inhibits STAT5 phosphorylation, and reduces NK cell killing in the presence of IFN-*γ* (56).

### Context-Dependent Role of Interleukin-18 in Cancer

Upon interaction with its heterodimeric receptor and the activation of the MyD88 signaling pathway, IL-18 primes NK cells to produce IFN-*γ* (104). *In vitro* data also show that IL-18 can favor the differentiation of human CD56^dim^ CCR7^+^ CD25^+^ CD83^+^ helper NK cells, which control tumor dissemination and CD8^+^ T cell activation through the crosstalk with DCs in the lymph nodes (40, 41, 105). Similar to other activating cytokines, such as IL-17 above, also IL-18 can display immunosuppressive features by boosting TGF-β-mediated immunosuppression (106), formation of MDSCs, and induction of PD1 expression on NK cells in mouse models (42, 107, 108). Notably, the upregulation of IL-18 binding protein (IL-18BP), which sequesters IL-18 as a physiological negative feedback mechanism, has been reported as an immune escape strategy (42, 109).

## Natural Killer Cell Dysfunction in the Tumor Microenvironment Triggered by Myeloid-Derived Cytokines

Although the NK-myeloid crosstalk stimulates anti-tumor immunity by NK cells in the early tumor development, immunosuppressive cytokines promoting NK cell dysfunction predominate in the aggravated TME of more advanced tumors. Tumor-associated macrophages (TAMs) and MDSCs are usually the main myeloid cell populations in such TME and represent the major producers of NK cell suppressive TGF-β and IL-10 (**Figure 1A** and **Table 1A**) (110).

### Transforming Growth Factor-β

As mentioned above, TGF-β is a key suppressor of NK cell migration, cytotoxicity, and cytokine production *via* transcriptional and post-transcriptional control of receptor and effector molecule expression (111). As an example, TGF-β-mediated downregulation of CX3CR1 can limit NK cell migration towards the tumor site (25); similarly, the downregulation of activating receptors including NKG2D and NKp30 as well as the adaptor proteins DAP10 and DAP12 triggered by TGF-β diminishes human NK cell cytotoxicity *in vitro* (26–28, 111). TGF-β-mediated NK cell conversion into Eomes^-^ ILC1 with increased expression levels of inhibitory receptors may represent an additional mechanism to reduce NK cell cytotoxicity in mouse tumor models (29), while a third mechanism is the inhibition of signaling pathways downstream of pro-inflammatory cytokines (30, 36, 111, 112). TGF-β-induced miRNA targets STAT1, which is essential for perforin expression (111), whereas blockade of IL-15-mediated mTOR activity dampens NK cell metabolism. Beyond this, TGF-β also reduces NK cell-mediated IFN-γ and TNF-α production in both human and mouse (29, 30, 36, 111, 112).

### Interleukin-10

Like TGF-β, IL-10 directly inhibits IFN-γ and TNF-α production by NK cells *in vitro*. This effect is also indirectly mediated *via* inhibition of IL-12, IL-15, and IL-18 production in myeloid cells (24, 58). Yet, in the presence of IL-12 and IL-18, IL-10 has also been shown to stimulate NK cell proliferation, cytotoxicity, and IFN-γ production *in vitro via* the STAT3 signaling pathway (24, 31, 113, 114). More studies are needed to determine this complex relationship between NK cell suppressive and possibly NK cell promoting properties of IL-10.

### Interleukin-32α

Another myeloid-derived immunosuppressive cytokine that can characterize the NK cell suppressive TME is IL-32α. IL-32α, which is often found highly expressed in the TME (115), inhibits IL-15-induced upregulation of perforin and granzyme B *in vitro* (34). Interestingly, dysregulated levels of IL-32α impairs human NK cell functions in chronic myelomonocytic leukemia and myelodysplastic syndrome (116).

### Interleukin-1β

IL-1β is released by monocytes, DCs, and macrophages and stimulates the expansion of CD11b^+^Gr-1^+^Ly6C^-^ MDSCs, which are potent inhibitors of murine NK cells *in vivo* (117). This cytokine has also been reported to maintain human NK cells in an immature state in the presence of IL-15 in secondary lymphoid tissues (23). Similarily to several cytokines above, IL-1β can also have NK cell promoting effects. One example is by indirectly promoting NK cell IFN-*γ* release by inducing IL-21 production in Th9 cells in mice (118).

## Natural Killer Cell-Derived Cytokines Regulating Myeloid Cells in the Tumor Microenvironment

Upon cytokine stimulation and target cell encounter, NK cells themselves produce a range of cytokines such as IFN-γ, TNF-α, granulocyte-macrophage colony-stimulating factor (GM-CSF), and in some cases IL-10, that in turn modulate myeloid cells (119–121) (**Figure 1A** and **Table 1B**). In this section, we will summarize how cytokines released by NK cells affect the NK-myeloid cell crosstalk.

### Dendritic Cells

DCs are central in triggering immune responses by T and NK cells. However, NK cells are also important for the DC function. In addition to cell-to-cell contact, NK cell-derived IFN-γ, TNF-α, and GM-CSF play a key role in the maturation and activation of human antigen-presenting DCs in the TME and lymphoid organs (59–61, 122). Further, activated human DCs maintain the IFN-γ production and induce high mobility group box 1 (HMGB1) production in NK cells *via* secretion of IL-12 and IL-18, resulting in further DC activation and maturation (43, 65). Hence, NK cells can be central in promoting DC maturation and activation, thereby feeding the positive loop of NK-myeloid crosstalk involving DCs in the TME.

### Tumor-Associated Macrophages

TAMs are often immunosuppressive and known drivers of tumor progression (123). However, macrophages in the TME have a high degree of plasticity and can even display a pro-inflammatory phenotype (M1) and release IFN-γ and TNF-α (124). *In vitro* studies have shown that IFN-γ is the main cytokine that drives classical activation of macrophages and polarizes them towards an M1-like phenotype (62). Indeed, as highlighted in a murine sarcoma model, NK cell-derived IFN-γ promotes M1 polarization of macrophages (63). Additionally, TNF-α, and GM-CSF also support an inflammatory phenotype in macrophages (62). Hence, NK cells can also have a critical role in maintaining a pro-inflammatory phenotype of TAMs.

### Tumor-Associated Neutrophils

Similar to TAMs, tumor-associated neutrophils (TANs) can be both tumor-promoting or pro-inflammatory and thereby counteract tumor growth. Recently, the first studies have investigated the interaction of NK cells with TANs in mouse models. NK cell-derived IFN-γ inhibits the tumor-promoting function of TANs in murine sarcoma and lung cancer models (64). NK cell-derived GM-CSF appears to promote Neutrophils Extracellular Trap formation by neutrophils, which can support tumor metastasis (66, 67).

### Myeloid-Derived Suppressor Cells

MDSCs include a heterogenous group of myeloid cells that have a markedly strong immunosuppressive ability. While NK cell-derived GM-CSF results in DC activation and macrophage polarization towards an anti-tumorigenic phenotype, GM-CSF expands MDSCs in human and murine tumors (62, 125, 126). The exact role of NK cell-derived GM-CSF in promoting MDSC expansion in the TME remains unclear but should likely not be neglected. Importantly, it is clear that NK cells can promote MDSCs in the TME also by other mechanisms. A CD73^+^ IL-10-secreting subset of NK cells was recently identified in human sarcomas (127). While IL-10 promotes regulatory T cells (Tregs), it has also been shown to maintain the immune-suppressive functions of MDSCs in ovarian cancer (128).

## Therapeutic Approaches to Directly Stimulate Natural Killer Cells or to Revert the Tumor Microenvironment to Favor Natural Killer Cell Anti-Tumor Responses

As highlighted in the previous sections, controlling the cytokine milieu in the TME is likely key to unleash the full potential of NK cell-based immunotherapies for several malignancies. Below, we will discuss ongoing and future approaches to enhance NK cell cytotoxicity in the TME (**Figure 1B**).

### Administration of Cytokines

Administration of cytokines to boost NK cell anti-tumor cytotoxicity has been widely explored in the recent years. IL-2, IL-12, IL-15, and IL-21 represent the most promising cytokines under investigation. While IL-2 acts on several immune cell populations, the effects of IL-15 is mainly limited to NK cells and CD8^+^ T cells. Promising results have been obtained in phase I clinical trials for melanoma and hematologic malignancies using ALT-803 (an IL-15 mutein/IL-15Rα complex fused with the IgG1 Fc) (129, 130). However, side-effects observed following high-dose administration of both IL-2 and IL-15 represents a major challenge along with mobilization of Tregs (131, 132). An alternative strategy to reduce the risk of side-effects while stimulating NK cells more specifically is the use of IL-2 mutants, such as IL-2 F42K and IL-2 H9 that compared to wildtype IL-2 preferentially bind IL-2Rα and thereby increases NK cell activation without inducing Treg expansion (133–135). Likewise, although the use of IL-18 therapy did not show toxicity and was ineffective in several clinical trials, a novel IL-18 mutant has been recently shown to induce NK cell anti-tumor activity (136).

To avoid the toxicities of high-dose cytokines, investigators have also addressed the use of lower doses following adoptive NK cell transfer or combined with checkpoint blockade or fused to anti-tumor antibodies. Intermediate doses of IL-2 have been explored to support adoptively infused NK cells (5). In this context, IL-2 is intended to promote persistence and expansion of the infused donor NK cells, however, data also show IL-2 mobilizes Tregs which likely counteract the effect of the transferred NK cells. As clinical protocols on adoptive NK cell transfer where post-infusion IL-2 has been omitted report similar outcomes as those with IL-2 (137), it remains unclear as to whether post-NK cell infusion IL-2 is of benefit or not. Administration of IL-15 has also been used to support adoptively infused NK cells. However, although in initial trials report this cytokine may trigger better responses, intermediate doses of IL-15 were associated with cytokine-release syndrome (CRS) when administrated subcutaneously (138). Additional investigations are needed to identify which cytokines and the window in which they promote the effect of adoptively infused NK cells.

IL-15, IL-12, and IL-21 are under clinical investigation combined with anti-CTLA4, anti-TIM3, or anti-PD-1 (139, 140). Alternative approaches that are also currently explored are based on the generation of anti-tumor antibody-cytokine fusion molecules. Trispecific killer engagers (TriKEs) fused with IL-15 have demonstrated their ability to boost NK cell functions and persistence (141, 142), whereas IL-21 fused to anti-CD20 increases mouse survival in a lymphoma model (143). Future studies have to address the clinical efficacy of these approaches.

### Modulation and Prevention of Cytokine Signaling

In addition to the more direct cytokine-based treatment approaches discussed above, there are also indirect strategies explored. Targeting pathways downstream of cytokine receptors may represent an alternative or complementary approach. Inhibition of SOCS proteins is promising since blockade of the STAT5 inhibitor CIS increases NK cell-mediated anti-tumor activity (144). In preclinical models, agonists of the stimulator of IFN genes (STING) pathway induce tumor regression by stimulating IL-15 production by infiltrating myeloid cells (145). Data also support the potential of GSK3 inhibitors in promoting maturation and cytotoxicity of NK cells following expansion *ex vivo* with IL-15 (146) GSK3 inhibition increased NK cell production of TNF and IFN-γ as well as bolstered the NK cytotoxicity *per se* and *via* ADCC, which translated into better tumor control of human ovarian cancer in a mouse model. An alterantive strategy is to alter the cytokine environment in the TME by neutralizing immunosuppressive cytokines. Ongoing trials with anti-TGF-β (Fresolimumab) and inhibitors of TGF-β signaling (140) will show if such approach has potential for the future. Depletion and/or prevention of the infiltration of suppressive cytokine-producing myeloid cells in the TME *per se* represents a tempting and yet incompletely explored alternative that indirectly would bolster the anti-tumor properties of NK cells. This approach needs further attention in future studies.

## Concluding Remarks

The network of cells and signals in the TME is complex and not yet fully understood. However, ample evidence show that this environment most often is NK cell suppressive, especially in more advanced disease. Yet, the NK-myeloid cell crosstalk is central in shaping NK cell anti-tumor responses and that a better understanding of this crosstalk is required to improve outcomes of NK cell-based cancer immunotherapies. While strategies directed towards boosting NK cell cytotoxicity *per se* using cytokines or drugs that modulate cytokine signaling, other complementory approaches directed towards reverting the TME to favor anti-tumor immunity is likely required to promote long-term responses.

As pointed out in this mini review, challenges hindering prompt progress in the field include the multitude of different cell types in the TME along with the context-dependent functions of several cytokines derived from both the myeloid cells and the NK cells. We predict that recent developments related to genetic engineering of NK cells along with the arsenal of new cytokine mutants as well as targeted and immunomodulatory drugs, alone or combination, can facilitate progress in the field. We foresee the use of genetically engineered NK cells to help improve their efficacy *per se* but also to resist and persist in the TME and thereby have the chance to revert it to a more pro-inflammatory milieu optimal for initating potent durable anti-tumor immune reponses. CAR-NK cells equipped with cytokine signaling elements or dominant negative cytokine receptors are examples that hold promise. We are positive that this along with further insights in the basic biology of cytokines and cytokine signaling will help improve NK cell-based cancer immunotherapy.

## Author Contributions

SG, KW, MC, and SM have together outlined and written the manuscript. All authors contributed to the article and approved the submitted version.

## Funding

This work was supported by funding from Jeanssons Stiftelser (MC), Swedish Society for Medical Research (MC), Wallenberg Clinical Fellow (MC), Cancerfonden (MC) and the Swedish Research Council (MC) as well as from Fondation Arc Pour La Recherche Sur Le Cancer (SM), Ligue Nationale Contre Le Cancer (SM), Cancéropôle Nord-Ouest (SM), and CPER (SM).

## Conflict of Interest

The authors declare that the research was conducted in the absence of any commercial or financial relationships that could be construed as a potential conflict of interest.
